# De-escalation of radiation therapy in patients with stage I, node-negative, HER2-positive breast cancer

**DOI:** 10.1038/s41523-021-00242-8

**Published:** 2021-03-25

**Authors:** Jose G. Bazan, Sachin R. Jhawar, Daniel Stover, Ko Un Park, Sasha Beyer, Erin Healy, Julia R. White

**Affiliations:** 1 Department of Radiation Oncology, The Ohio State University Comprehensive Cancer Center—Arthur G. James Cancer Hospital and Solove Research Institute, Stefanie Spielman Comprehensive Breast Center, Columbus, OH USA; 2 Department of Internal Medicine, Division of Medical Oncology, The Ohio State University Comprehensive Cancer Center—Arthur G. James Cancer Hospital and Solove Research Institute, Stefanie Spielman Comprehensive Breast Center, Columbus, OH USA; 3 Department of Surgical Oncology, The Ohio State University Comprehensive Cancer Center—Arthur G. James Cancer Hospital and Solove Research Institute, Stefanie Spielman Comprehensive Breast Center, Columbus, OH USA

**Keywords:** Breast cancer, Radiotherapy

## Abstract

In the modern era, highly effective anti-HER2 therapy is associated with low local-regional recurrence (LRR) rates for early-stage HER2+ breast cancer raising the question of whether local therapy de-escalation by radiation omission is possible in patients with small-node negative tumors treated with lumpectomy. To evaluate existing data on radiation omission, we used the National Cancer Database (NCDB) to test the hypothesis that RT omission results in equivalent overall survival (OS) in stage 1 (T1N0) HER2+ breast cancer. We excluded patients that received neoadjuvant systemic therapy. We stratified the cohort by receipt of adjuvant radiation. We identified 6897 patients (6388 RT; 509 no RT). Patients that did not receive radiation tended to be ≥70 years-old (odds ratio [OR] = 3.69, 95% CI: 3.02–4.51, *p* < 0.0001), to have ≥1 comorbidity (OR = 1.33, 95% CI: 1.06–1.68, *p* = 0.0154), to be Hispanic (OR = 1.49, 95% CI: 1.00–2.22, *p* = 0.049), and to live in lower income areas (OR = 1.32, 95% CI: 1.07–1.64, *p* = 0.0266). Radiation omission was associated with a 3.67-fold (95% CI: 2.23–6.02, *p* < 0.0001) increased risk of death. While other selection biases that influence radiation omission likely persist, these data should give caution to radiation omission in T1N0 HER2+ breast cancer.

## Introduction

Breast conservation therapy with lumpectomy and breast irradiation results in equivalent cancer control outcomes to mastectomy for patients with early stage breast cancer (BC) on the basis ofs randomized controlled clinical trials^1–4^. Subsequent efforts have focused on de-escalation of breast irradiation by reducing treatment duration with hypofractionated radiation schedules^5–8^ or reducing treatment volume with accelerated partial breast irradiation (APBI)^9–12^. Consensus guidelines now recommend hypofractoinated breast RT for nearly all patients with stage I–II, node-negative BC after lumpectomy^13^ and APBI is an appropriate option for patients with stage I, node-negative ER+ BC based on the results of the two largest APBI trials^11,12^.

However, the ultimate form of de-escalation is radiation omission. Randomized trials have shown that use of endocrine therapy and radiation omission in elderly women with stage I, node-negative, ER+ BC results in higher rates of ipsilateral breast tumor recurrences without a resulting increased risk of distant metastases or BC mortality^14,15^. Several prospective studies are evaluating RT omission in younger women (<65 years old) with stage I, ER+/HER2−, node-negative BC that are low-risk by genomic assay^16–21^.

To date, no studies have prospectively evaluated RT omission in HER2+ BC treated with upfront surgery. Prior to the use of anti-HER2 directed therapies, local-regional recurrence (LRR) rates were high in patients with HER2+ BC but have s decreased with the use of trastuzamab and other anti-HER2 directed therapies^22^. As an attempt to de-escalate systemic therapy for HER2+ BC, the adjuvant paclitaxel trastuzamab (APT) trial evaluated upfront surgery (mastectomy or lumpectomy) followed by adjuvant paclitaxel + trastuzumab for 12 weeks and trastuzumab for a total of 1 year in patients with T1-2N0-N1mic HER2+ BC^23,24^. The trial population was predominantly stage I, HER2+ BC as fewer than 10% had T2 tumors and only 1% had microscopic nodal disease. Results demonstrated a 7-year LRR-free survival rate of 98.6% for all patients and 99.0% in the 217 patients treated with lumpectomy+radiation^25^. In addition to the results from the APT trial, preliminary findings from a randomized phase II study of adjuvant ado-trastuzumab emtansine (TDM-1) vs. trastuzumab + paclitaxel in patients with stage I HER2+ BC showed excellent local-regional control with only 2 LRR events out of 383 patients in the TDM-1 group and 4 LRR events in the 114 patients treated with paclitaxel-trastuzmab^26^. These exceedingly low LRR rates suggests that investigation of RT omission may be warranted in patients with T1N0 HER2+ BC treated with upfront lumpectomy and adjuvant trastuzumab-based systemic therapy.

Here, we evaluate the existing data on radiation omission in HER2+ BC by using the National Cancer Database (NCDB) to test that hypothesis that RT omission results in equivalent overall survival (OS) in stage I (T1N0) HER2+ BC treated with lumpectomy and adjuvant trastuzumab-based systemic therapy.

## Results

### Patient characteristics

**Fig. 1:**
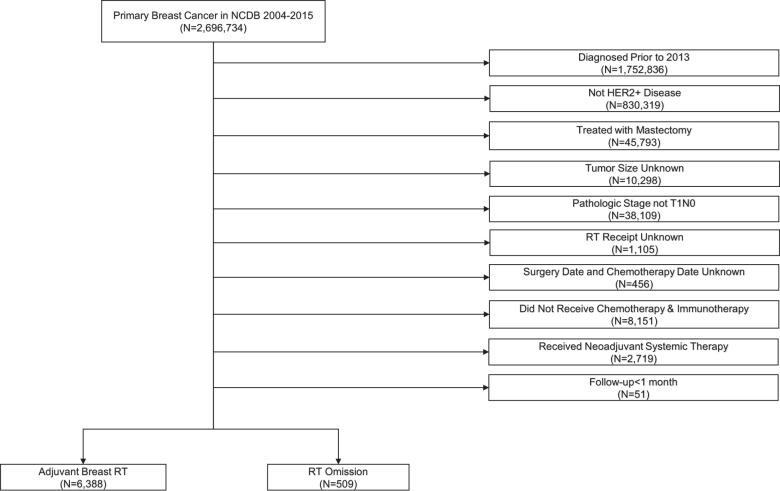
Study flow diagram for analytic cohorts. NCDB National Cancer Database, RT radiation therapy.

Figure 1 demonstrates that 6897 patients met the inclusion criteria with 509 (7.4%) in the radiation omission group and 6388 (92.6%) in the radiation group. More than 75% of patients had hormone-sensitive disease, >60% had T1c tumors and ~50% had grade 3 disease (Table 1). Patients treated with radiation mostly received conventionally fractionated radiation therapy (63.9%), followed by hypofractionated radiation (23.7%), unknown regimen due to lack of specific details (11.7%), and 0.7% received APBI. In the 509 patients in the omission group, radiation was recommended but not delivered for patient refusal or unknown reasons in 254 patients; radiation was not planned as part of the treatment course in 234 patients; and radiation was contraindicated in 21 patients. We found no difference in patient- or tumor-related characteristics between the 234 patients in which radiation was not planned compared to the 254 patients that refused radiation (Supplementary Table 1). Adjuvant chemotherapy was classified as single-agent in 2341 patients (33.9%), multi-agent in 4449 (64.5%), and not specified in 107 patients (1.6%). Endocrine therapy was administered in 86% (*N* = 4520) of the hormone-sensitive patients (88.8% radiation group vs. 47.8% radiation omission group, *p* < 0.0001).

**Table 1:** Baseline patient characteristics in the entire cohort and by receipt of radiation therapy.

	Entire Cohort (*N* = 6897)	Had RT (*N* = 6388)	RT omission (*N* = 509)	*p* Value
Age				
Mean (standard deviation)	59.6 (10.4)	59.2 (10.2)	64.0 (11.6)	<0.0001
≥70 yrs, *N* (%)	1204 (17.5%)	996 (15.6%)	208 (40.9%)	<0.0001
Comorbidities				<0.0001
0	5845 (84.8%)	5445 (85.2%)	400 (78.6%)	
≥1	1052 (15.2%)	943 (14.8%)	109 (21.4%)	
Laterality				0.1962
Left	3564 (51.7%)	3315 (51.9%)	249 (48.9%)	
Right	3333 (48.3%)	3073 (48.1%)	260 (51.1%)	
Hormone status				0.4174
ER+ or PR+	5264 (76.3%)	4883 (76.4%)	381 (74.9%)	
ER−/PR−	1633 (23.7%)	1505 (23.6%)	128 (25.1%)	
Tumor size				0.2204
≤1 cm	2504 (36.3%)	2332 (36.5%)	172 (33.8%)	
>1–2 cm	4393 (63.7%)	4056 (63.5%)	337 (66.2%)	
Tumor grade				0.3196
Grade 3	3346 (48.5%)	3088 (48.3%)	258 (50.7%)	
Grade 1–2	3277 (47.5%)	3050 (47.8%)	227 (44.6%)	
Unknown	274 (4.0%)	250 (3.9%)	24 (8.8%)	
Facility type				0.3496
Academic	2224 (32.3%)	2074 (32.5%)	150 (29.5%)	
Other	4499 (65.2%)	4152 (65.0%)	347 (7.7%)	
Unknown	174 (2.5%)	162 (2.5%)	12 (6.9%)	
Race/ethnicity				0.3428
Non-Hispanic White	5459 (79.2%)	5063 (79.3%)	396 (77.8%)	
Non-Hispanic Black	765 (11.1%)	714 (11.2%)	51 (10.0%)	
Hispanic	331 (4.8%)	300 (4.7%)	31 (6.1%)	
Non-Hispanic Other	299 (4.3%)	273 (4.2%)	26 (5.1%)	
Unknown	43 (0.6%)	38 (0.6%)	5 (1.0%)	
Income status				0.0016
<$46,000/yr	3432 (49.8%)	3145 (49.2%)	287 (56.4%)	
≥$46,000/yr	3269 (47.4%)	3066 (48.0%)	203 (39.9%)	
Unknown	196 (2.8%)	177 (2.8%)	19 (3.7%)	
No HSD				0.0467
≥20%	2196 (31.8%)	2014 (31.5%)	182 (35.8%)	
<20%	4505 (65.3%)	4197 (65.7%)	308 (60.5%)	
Unknown	196 (2.8%)	177 (2.8%)	19 (3.7%)	
Distance to facility				0.8743
≥8.9 miles	3465 (50.2%)	3211 (50.3%)	254 (49.9%)	
<8.9 miles	3432 (49.8%)	3177 (49.7%)	255 (50.1%)	
Chemotherapy				0.0022
Single-agent	2341 (33.9%)	2140 (33.5%)	201 (39.5%)	
Multi-agent	4449 (64.5%)	4154 (65.0%)	295 (58.0%)	
Not specified	107 (1.6%)	94 (1.5%)	13 (2.5%)	
Receipt of ET^a^				<0.0001
Yes	4520 (85.9%)	4338 (88.8%)	182 (47.8%)	
No	744 (14.1%)	545 (11.2%)	199 (52.2%)	

*RT* radiation therapy, *NHW* non-Hispanic white, *NHB* non-Hispanic Black, *HSD* high-school diploma.

^a^Only in patients with ER+ and/or PR+ disease.

### Factors associated with radiation omission

Patients that did not receive radiation tended to be older (mean age = 64.0 years [standard deviation (SD) = 11.6] vs. 59.2 years [SD = 10.2], *p* < 0.0001 and proportion ≥ 70 years-old vs. <70 years-old 40.9% vs. 15.6%, *p* < 0.0001) and were more likely to have at least 1 comorbidity (21.4% vs. 14.8%, *p* < 0.0001). In addition, Table 1 shows that patients in the radiation omission group tended to live in areas with median household income <$46,000 (56.4% vs. 49.2%, *p* = 0.0016) and in areas where at least 20% of people did not have high school diplomas (35.8% vs. 31.5%, *p* = 0.0467).

On multivariate logistic regression analysis, Table 2 demonstrates that age ≥ 70 (OR = 3.69, 95% CI: 3.02–4.51, *p* < 0.0001), presence of ≥1 comorbidity (OR = 1.33, 95% CI: 1.06–1.68, *p* = 0.0154), Hispanic ethnicity (OR = 1.49, 95% CI: 1.00–2.22, *p* = 0.049), and living in an area with household income < $46,000 (OR = 1.32, 95% CI: 1.07–1.64, *p* = 0.0266) were all independently associated with increased odds of radiation omission.

**Table 2:** Logistic regression analysis for factors associated with omission of radiation therapy. An odds ratio >1 implies that factor is associated with omission of RT while odds ratio <1 implies the factor is associated with receipt of RT.

	Univariate analysis [OR, 95% CI, *p* value]	Multivariate analysis [OR, 95% CI, *p* value]
Age		
≥70 yrs vs. <70	3.74 (3.10–4.52), *p* < 0.0001	3.69 (3.02–4.51), *p* < 0.0001
Comorbidities		
0	Reference	Reference
≥1	1.57 (1.26–1.97), *p* < 0.0001	1.33 (1.06–1.68), *p* = 0.0154
Laterality		
Right	Reference	Reference
Left	0.89 (0.74–1.06), *p* = 0.1964	0.88 (0.73–1.06), *p* = 0.1737
Hormone status		
ER+ or PR+	Reference	N/A
ER−/PR−	1.09 (0.885–1.34), *p* = 0.4176	
Tumor size		
≤1 cm	Reference	N/A
>1–2 cm	1.13 (0.93–1.36), *p* = 0.2206	
Tumor grade		
Grade 1–2	Reference	N/A
Grade 3	1.12 (0.93–1.35), *p* = 0.2212	
Unknown	1.29 (0.83–2.00), *p* = 0.2567	
Facility type		
Other	Reference	Reference
Academic	0.87 (0.71–1.06), *p* = 0.1537	0.96 (0.78–1.18), *p* = 0.6649
Unknown	0.89 (0.49–1.61), *p* = 0.6918	1.29 (0.71–2.37), *p* = 0.4051
Race/ethnicity		
NH-White	Reference	Reference
NH-Black	0.91 (0.68–1.24), *p* = 0.5558	0.96 (0.70–1.31), *p* = 0.7763
Hispanic	1.32 (0.90–1.94), *p* = 0.1542	1.49 (1.00–2.22), *p* = 0.0498
NH-Other	1.22 (0.80–1.84), *p* = 0.2143	1.46 (0.95–2.24), *p* = 0.0813
Unknown	1.68 (0.66–4.30), *p* = 0.2771	2.00 (0.77–5.23), *p* = 0.1560
No HSD		
<20%	Reference	Reference
≥20%	1.23 (1.02–1.49), *p* = 0.0325	1.07 (0.85–1.34), *p* = 0.5881
Unknown	1.46 (0.90–2.38), *p* = 0.1252	1.69 (1.02–2.80), *p* = 0.0424
Income status		
≥$46,000/yr	Reference	Reference
<$46,000/yr	1.38 (1.14–1.66), *p* = 0.0007	1.32 (1.07–1.64), *p* = 0.0266
Unknown	1.62 (0.99–2.66), *p* = 0.0549	1.68 (1.01–2.79), *p* = 0.0443
Distance to facility		N/A
<8.9 miles	Reference	
≥8.9 miles	0.99 (0.82–1.18), *p* = 0.8743	
Chemotherapy		
Single-agent	Reference	Reference
Multi-agent	0.76 (0.63–0.91), *p* = 0.0033	0.96 (0.79–1.17), *p* = 0.6878
Not specified	1.47 (0.81–2.68), *p* = 0.2045	1.49 (0.81–2.76), *p* = 0.2044

*OR* odds ratio, *CI* confidence interval, *NHW* non-Hispanic white, *NHB* non-Hispanic Black, *HSD* high-school diploma.

### Survival outcomes by receipt of radiation

Median follow-up was 29.4 months (IQR: 19.5–39.9 months) with 155 deaths (60 radiation omission; 95 radiation received). The 2-year OS was significantly worse for patients with radiation omission (88.9% vs. 99.2%, *p* < 0.0001). Hormone-receptor status (ER−/PR− vs. hormone-sensitive, HR = 1.01 [95% CI: 0.7–1.46], *p* = 0.95), tumor size (*p* = 0.14) and grade 3 tumors (*p* = 0.17) were not significantly associated with OS on univariate analysis. However, when factoring in receipt of endocrine therapy with hormone-receptor status, patients with hormone-sensitive disease that did not receive endocrine therapy (HR = 5.55, 95% CI: 3.86–7.97, *p* < 0.0001) and patients with ER−/PR− disease (HR = 1.60, 95% CI: 1.07–2.40, *p* = 0.0222) were associated with worse OS compared to patients with hormone-sensitive disease that received endocrine therapy. Table 3 demonstrates that the adjusted HR for OS in the radiation omission group was 5.32 (95% CI: 3.68–7.69, *p* < 0.0001). Age ≥ 70 (HR = 2.03 95% CI: 1.42–2.88, *p* < 0.0001), patients with hormone-sensitive disease that did not receive endocrine therapy (HR = 3.33, 95% CI: 2.23–5.00, *p* < 0.0001), and tumor size > 1 cm (HR = 1.45, 95% CI: 1.01–2.07, *p* = 0.0438) were independently associated with worse OS while there was a trend toward an association with worse OS in patients with ≥1 comorbidity and those with grade 3 disease and an association with improved OS in patients of Other (non-Hispanic/Non-Black/non-White) race and those that received multi-agent chemotherapy.

**Table 3:** Cox regression analysis of overall survival for the entire patient cohort.

	Univariate analysis [HR, 95% CI, *p* value]	Multivariate analysis [HR, 95% CI, *p* value]
No RT vs. RT	10.18 (7.36–14.08), *p* < 0.0001	5.32 (3.68–7.69), *p* < 0.0001
Age		
≥70 yrs vs. <70	3.69 (2.68–5.09), *p* < 0.0001	2.03 (1.42–2.88), *p* < 0.0001
Comorbidities		
0	Reference	Reference
≥1	2.00 (1.40–2.87), *p* = 0.0002	1.41 (0.97–2.05), *p* = 0.0716
Laterality		
Right	Reference	N/A
Left	0.93 (0.68–1.28), *p* = 0.6590	
Hormone status		
ER+ or PR+	Reference	N/A
ER−/PR−	1.01 (0.70–1.46), *p* = 0.9488	
Treatment group		
HS, received ET	Reference	Reference
HS, no ET	5.55 (3.86–7.97), *p* < 0.0001	3.33 (2.23–5.00), *p* < 0.0001
ER−/PR−	1.60 (1.07–2.40), *p* = 0.0222	1.28 (0.84–1.95), *p* = 0.2490
Tumor size		
≤1 cm	Reference	Reference
>1–2 cm	1.29 (0.92–1.82), *p* = 0.1418	1.45 (1.01–2.07), *p* = 0.0438
Tumor grade		
Grade 1–2	Reference	Reference
Grade 3	1.25 (0.91–1.73), *p* = 0.1730	1.33 (0.95–1.87), *p* = 0.0985
Unknown	0.87 (0.38–2.02), *p* = 0.7507	1.01 (0.43–2.35), *p* = 0.9841
Facility type		
Other	Reference	Reference
Academic	0.75 (0.53–1.07), *p* = 0.1077	0.80 (0.56–1.15), *p* = 0.2363
Unknown	0.21 (0.03–1.48), *p* = 0.1166	0.22 (0.03–1.62), *p* = 0.1385
Race/ethnicity		
NH-White	Reference	Reference
NH-Black	1.16 (0.72–1.86), *p* = 0.5368	1.19 (0.73–1.96), *p* = 0.4849
Hispanic	0.82 (0.36–1.85), *p* = 0.6290	0.73 (0.32–1.68), *p* = 0.4578
NH-Other	0.15 (0.02–1.04), *p* = 0.0547	0.14 (0.02–1.02), *p* = 0.0526
Unknown	0.97 (0.14–6.96), *p* = 0.9782	0.87 (0.12–6.26), *p* = 0.8873
No HSD		
<20%	Reference	Reference
≥20%	1.38 (0.994–1.91), *p* = 0.0546	1.22 (0.80–1.76), *p* = 0.3858
Unknown	0.78 (0.25–2.45), *p* = 0.6664	0.59 (0.23–2.40), *p* = 0.6235
Income status		
≥$46,000/yr	Reference	Reference
<$46,000/yr	1.50 (1.08–2.07), *p* = 0.0149	1.21 (0.82–1.78), *p* = 0.4444
Unknown	0.87 (0.27–2.76), *p* = 0.8097	0.90 (0.28–2.88), *p* = 0.8627
Distance to facility		
<8.9 miles	Reference	N/A
≥8.9 miles	0.89 (0.65–1.21), *p* = 0.4489	
Chemotherapy		
Single-agent	Reference	Reference
Multi-agent	0.61 (0.44–0.85), *p* = 0.0036	0.72 (0.51–1.01), *p* = 0.0593
Not specified	0.99 (0.31–3.17), *p* = 0.9918	1.01 (0.32–3.25), *p* = 0.9830

Hazards ratio > 1 implies the factor is associated with increased risk of death.

*HR* hazards ratio, *CI* confidence interval, *RT* radiation therapy, *HS* hormone-sensitive, *ET* endocrine therapy, *NHW* non-Hispanic white, *NHB* non-Hispanic Black, *HSD* high-school diploma.

We also performed a subgroup analysis by hormone-receptor status. Table 4 demonstrates the multivariate results for patients with ER−/PR− disease and for hormone-sensitive disease. Radiation omission was associated with a 4.2-fold increased risk of death in the ER−/PR− cohort and a 5.7-fold increased risk of death in the hormone-sensitive cohort. Endocrine therapy omission was also independently associated with an approximate threefold increased risk of death in the hormone-sensitive cohort.

**Table 4:** Multivariate Cox regression analysis for overall survival by hormone-receptor status. Hazards ratio > 1 implies the factor is associated with increased risk of death.

	ER−/PR− (*N* = 1633) [HR, 95% CI, *p* value]	Hormone-sensitive (*N* = 5264) [HR, 95% CI, *p* value]
No RT vs. RT	4.20 (1.93–9.11), *p* = 0.0003	5.72 (3.73–8.76), *p* < 0.0001
No ET vs. ET	N/A	3.14 (2.07–4.77), *p* < 0.0001
Age		
≥70 yrs vs. <70	2.24 (1.07–4.66), *p* = 0.0317	2.01 (1.34–3.00), *p* = 0.0007
Comorbidities		
0	Reference	Reference
≥1	1.09 (0.48–2.50), *p* = 0.8389	1.57 (1.07–2.46), *p* = 0.0225
Tumor size		
≤1 cm	Reference	Reference
>1–2 cm	1.40 (0.68–2.88), *p* = 0.3556	1.51 (0.94–2.13), *p* = 0.0969
Tumor grade		
Grade 1–2	Reference	Reference
Grade 3	1.13 (0.53–2.42), *p* = 0.7569	1.33 (0.92–1.94), *p* = 0.1319
Unknown	0.59 (0.07–4.78), *p* = 0.6231	1.20 (0.48–3.02), *p* = 0.7013
Facility type		
Other	Reference	Reference
Academic	0.86 (0.42–1.75), *p* = 0.6709	0.76 (0.50–1.72), *p* = 0.1862
Unknown	0.00 (0.00–0.00), *p* = 0.9862	0.23 (0.03–1.72), *p* = 0.1530
No HSD		
<20%	Reference	Reference
≥20%	1.66 (0.72–3.82), *p* = 0.2355	1.15 (0.74–1.77), *p* = 0.5349
Unknown	1.20 (0.16–9.14), *p* = 0.8604	0.47 (0.11–1.97), *p* = 0.2986
Income status		
≥$46,000/yr	Reference	Reference
<$46,000/yr	0.78 (0.34–1.79), *p* = 0.5553	1.41 (0.92–2.18), *p* = 0.1191
Unknown	1.15 (0.15–8.73), *p* = 0.8940	0.46 (0.11–1.94), *p* = 0.2914
Chemotherapy		
Single-agent	Reference	Reference
Multi-agent	0.76 (0.36–1.60), *p* = 0.4678	0.69 (0.47–1.02), *p* = 0.0655
Not specified	1.34 (0.17–10.61), *p* = 0.7792	0.93 (0.22–3.87), *p* = 0.9220

*HR* hazards ratio, *CI* confidence interval, *RT* radiation therapy, *ET* endocrine therapy, *HSD* high-school diploma.

**Fig. 2:**
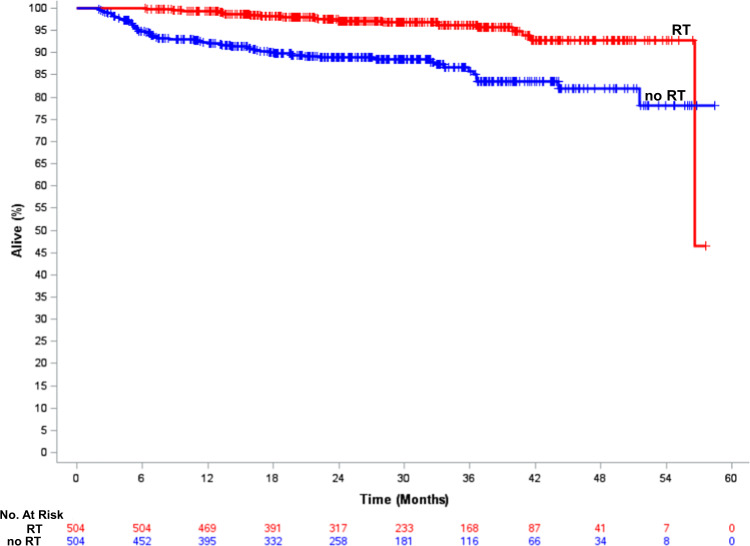
Overall survival of the propensity-score matched patients treated with breast radiation compared to those patients in which radiation was omitted. RT radiation therapy.

With propensity-score matching, we identified a radiation-treated match (using a caliper size of 0.178 based on a propensity score SD of 0.089) for 504 of 509 patients that did not receive RT. All baseline covariates were well-balanced based on a standardized difference of <0.10 (Table 5). Median follow-up for the 1008 patients was 26.5 months (IQR: 16.3–37.0 months) and there were 79 deaths (60 radiation omission; 19 radiation received). Figure 2 demonstrates that the 2-year OS in the propensity-matched cohort was 88.9% vs. 97.1% (HR = 3.67, 95% CI: 2.23–6.02, *p* < 0.0001).

**Table 5:** Characteristics of the propensity-matched cohort.

	Received RT (*N* = 504)	RT omission (*N* = 504)	Standardized difference
Age			
Mean (SD)	63.7 (11.0)	64.0 (11.6)	0.0340
≥70 yrs, *N* (%)	207 (41.1%)	204 (40.5%)	0.0121
Comorbidities			0.0097
0	398 (79.0%)	396 (78.6%)	
≥1	106 (21.0%)	108 (21.4%)	
Laterality			0.0596
Left	261 (51.8%)	246 (48.8%)	
Right	243 (48.2%)	258 (51.2%)	
Hormone status			0.0276
ER+ or PR+	382 (75.8%)	376 (74.6%)	
ER−/PR−	122 (24.2%)	128 (25.4%)	
Treatment group			0.0309
HS and received ET	188 (37.3%)	182 (36.1%)	
HS, no ET	194 (38.5%)	194 (38.5%)	
ER−/PR−	122 (24.2%)	128 (25.4%)	
Tumor size			0.0594
≤1 cm	156 (31.0%)	170 (33.7%)	
>1–2 cm	348 (69.0%)	334 (66.3%)	
Tumor grade			0.0122
Grade 3	260 (51.6%)	257 (51.0%)	
Grade 1–2	221 (43.9%)	224 (44.5%)	
Unknown	23 (4.5%)	23 (4.5%)	
Facility type			0.0281
Academic	147 (29.2%)	150 (29.8%)	
Other	344 (68.3%)	343 (68.1%)	
Unknown	13 (2.5%)	11 (2.1%)	
Race/ethnicity			0.0734
NH-White	407 (80.7%)	393 (78.0%)	
NH-Black	43 (8.5%)	50 (9.9%)	
Hispanic	25 (5.0%)	30 (6.0%)	
NH-Other	25 (5.0%)	26 (5.1%)	
Unknown	4 (0.8%)	5 (1.0%)	
Income status			0.0082
<$46,000/yr	282 (56.0%)	284 (56.4%)	
≥$46,000/yr	204 (40.5%)	202 (40.1%)	
Unknown	18 (3.5%)	18 (3.5%)	
No HSD			0.0084
≥20%	178 (35.3%)	180 (35.7%)	
<20%	308 (61.1%)	306 (60.7%)	
Unknown	18 (3.6%)	18 (3.6%)	
Distance to facility			
≥8.9 miles	242 (48.0%)	251 (49.8%)	0.0357
<8.9 miles	262 (52.0%)	253 (50.2%)	
Chemotherapy			0.0787
Single-agent	213 (42.2%)	198 (39.3%)	
Multi-agent	283 (56.2%)	294 (58.3%)	
Not specified	8 (1.6%)	12 (2.4%)	

*RT* radiation therapy, *SD* standard deviation, *HS* hormone-sensitive (ER+ and/or PR+), *ET* endocrine therapy, *NHW* non-Hispanic white, *NHB* non-Hispanic Black, *HSD* high-school diploma.

### Survival outcomes in patients that did not receive radiation therapy

In the 509 patients that did not receive radiation therapy, Supplementary Fig. 1 shows that hormone-sensitive patients that did not receive endocrine therapy had the worse outcomes with 2-yr OS rate of 83.0% compared to 93.8% in hormone-sensitive patients that received endocrine therapy and 91.2% in ER−/PR− patients. Table 6 demonstrates that on univariate and multivariate analysis, the only factor significantly associated with improved OS in the radiation omission cohort was receptor status with HR = 0.33 (95% CI: 0.18–0.61) for patients with hormone-sensitive disease treated with endocrine therapy relative to patients with hormone-sensitive disease that did not receive endocrine therapy and HR = 0.35 (95% CI: 0.17–0.70) for ER−/PR− patients relative to hormone-sensitive patients that did not receive endocrine therapy. No other patient- or tumor-related factors were associated with OS.

**Table 6:** Cox regression analysis of overall survival for the patients that did not receive radiation therapy.

	Univariate analysis [HR, 95% CI, *p* value]	Multivariate analysis [HR, 95% CI, *p* value]
Age		
≥70 yrs vs. <70	1.41 (0.85–2.33), *p* = 0.1876	1.23 (0.73–2.07), *p* = 0.4352
Comorbidities		
0	Reference	Reference
≥1	1.47 (0.84–2.58), *p* = 0.1800	1.48 (0.83–2.63), *p* = 0.1878
Laterality		
Right	Reference	N/A
Left	1.13 (0.68–1.87), *p* = 0.6388	
Hormone status		
HS, no ET	Reference	Reference
HS, received ET	0.37 (0.20–0.69), *p* = 0.0018	0.33 (0.18–0.61), *p* = 0.0005
ER−/PR−	0.40 (0.20–0.81), *p* = 0.0111	0.35 (0.17–0.70), *p* = 0.0032
Tumor size		
≤1 cm	Reference	N/A
>1–2 cm	1.04 (0.60–1.79), *p* = 0.8894	
Tumor grade		
Grade 1–2	Reference	N/A
Grade 3	1.28 (0.76–2.15), *p* = 0.3597	
Unknown	0.86 (0.20–3.64), *p* = 0.8366	
Facility type		
Other	Reference	Reference
Academic	0.63 (0.34–1.16), *p* = 0.1404	0.64 (0.34–1.19), *p* = 0.1604
Unknown	0.00 (0.00-Inf), *p* = 0.9804	0.00 (0.00-Inf), *p* = 0.9917
Race/ethnicity		
NH-White	Reference	Reference
NH-Black	0.81 (0.32–2.03), *p* = 0.6543	0.81 (0.32–2.04), *p* = 0.6560
Hispanic	0.24 (0.03–1.73), *p* = 0.1565	0.23 (0.03–1.71), *p* = 0.1527
NH-Other	0.00 (0.00-Inf), *p* = 0.9829	0.00 (0.00–Inf), *p* = 0.9887
Unknown	1.37 (0.19–9.94), *p* = 0.7534	1.47 (0.20–10.86), *p* = 0.7089
No HSD		
<20%	Reference	N/A
≥20%	0.76 (0.43–1.32), *p* = 0.3213	
Unknown	0.40 (0.06–2.88), *p* = 0.3608	
Income status		
≥$46,000/yr	Reference	N/A
<$46,000/yr	1.03 (0.61–1.73), *p* = 0.9230	
Unknown	0.44 (0.06–3.27), *p* = 0.4245	
Distance to facility		N/A
<8.9 miles	Reference	
≥8.9 miles	0.93 (0.56–1.55), *p* = 0.7915	
Chemotherapy		
Single-agent	Reference	N/A
Multi-agent	0.99 (0.58–1.69), *p* = 0.9711	
Not specified	1.61 (0.38–6.83), *p* = 0.5216	

Hazards ratio > 1 implies the factor is associated with increased risk of death.

*HR* hazards ratio, *CI* confidence interval, *RT* radiation therapy, *HS* hormone-sensitive (ER+ and/or PR+), *ET* endocrine therapy, *NHW* non-Hispanic white, *NHB* non-Hispanic Black, *HSD* high-school diploma.

**Fig. 3:**
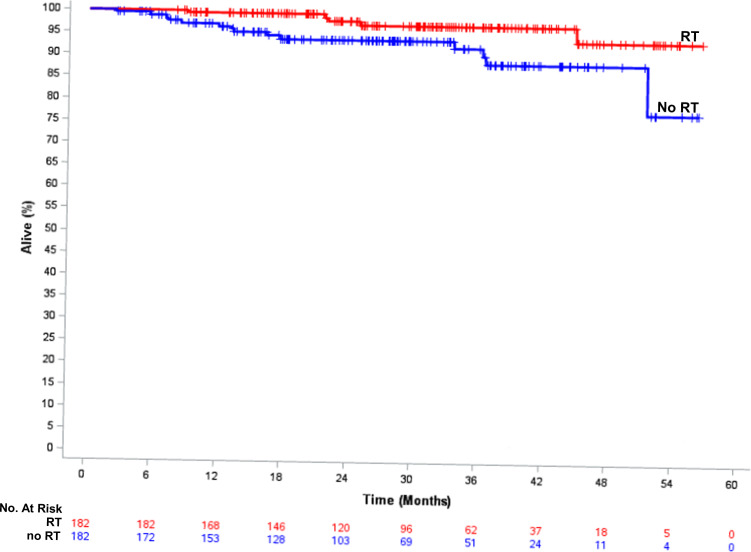
Overall survival of hormone-sensitive breast cancer patients that received endocrine therapy in a propensity-score matched cohort of those treated with breast radiation compared to those patients in which radiation was omitted. RT radiation therapy.

Since patients with T1N0 HER2+ and hormone-sensitive BC that receive endocrine therapy represent a low-risk group in which de-escalation of therapy may be considered, we performed a propensity-score matched analysis of OS based on receipt of radiation in this specific subgroup. As shown in Table 1, this subgroup represents 4520 patients, of which 182 did not receive radiation therapy. With propensity-score matching, we identified a radiation-treated match (using a caliper size of 0.007 based on a propensity score SD of 0.035) for 182 of 182 patients that did not receive RT. All baseline covariates were well-balanced based on a standardized difference of <0.10 with the exception of laterality which showed moderate balance with standardized difference = 0.12 (Supplementary Table 2). Median follow-up for the 364 patients was 28.1 months (IQR,: 18.1–38.0 months) and there were 19 deaths (14 radiation omission; 5 radiation received). Figure 3 demonstrates that the 2-year OS in the propensity-matched cohort was 93.8% vs. 97.9% (HR = 3.26, 95% CI: 1.17–9.10, *p* = 0.0240).

## Discussion

We sought to identify whether radiation omission has a measurable effect on overall survival in patients with T1N0 HER2+ BC treated with lumpectomy, adjuvant chemotherapy, and anti-HER2 therapy. We found that only a small proportion (~7%) of patients with T1N0 HER2+ BC treated with lumpectomy and adjuvant HER2-based systemic therapy did not receive adjuvant radiation in a large cohort of patients treated from 2013 to 2015. Even in this cohort of early stage patients treated with modern adjuvant systemic therapy, radiation omission was associated with significantly worse OS when adjusting for potential confounders and on propensity-score matched analysis. Factors associated with radiation omission included older age, presence of ≥1 comorbidity, Hispanic ethnicity, and living in an area with median household income <$46,000/year.

As cancer control outcomes for patients with BC have improved, de-escalation of local and systemic therapies in order to improve the therapeutic ratio has become a central theme of many prospective studies. The issue of omission of adjuvant radiation therapy for HER2+ BC was hypothesized several years ago by Dr. Bellon^27^, but radiation therapy continuously contributes, in a multidisciplinary context, to modify the natural history and management of the disease^28^. In particular, there have been numerous attempts at identifying which BC patients who undergo lumpectomy can safely omit adjuvant breast irradiation^29^. Multiple studies support that breast conservation without radiation looks promising for a subset of women with T1N0 ER+/HER2− BC^14,15,30–32^. To date, HER2+ BC has not been studied for de-escalation of breast irradiation post lumpectomy in part because of the high recurrence rates historically associated with HER2+ BC in the pre-trastuzumab era^33^. The publication of the APT trial that successfully de-escalated adjuvant chemotherapy intensity (no doxorubicin or cyclophosphamide) in early stage HER2+ BC has generated interest in de-escalating post-lumpectomy breast irradiation in the same population. Since the local recurrence events in the APT trial were infrequent, it is tempting to consider de-escalation of breast irradiation in this patient population. Unfortunately, there are scant data regarding recurrence rates for HER2+ BC post-lumpectomy in the absence of radiation. This is in contrast to T1N0 ER+/HER2− BC where an abundance of prior clinical trials and other data exists establishing that an acceptable hazard ratio for local recurrence can be expected in the absence of post-lumpectomy irradiation^34^.

The difference in OS between patients that received radiation and those that did not in this study was striking. It is notable that the survival curves separate early with a large difference in OS notable within the first 6 months of follow-up. This finding is inconsistent with the 2005 Early Breast Cancer Trialists’ Group meta-analysis that showed that reductions in BC mortality with adjuvant radiation were seen at 15 years and only in patients that had a >10% absolute reduction in LRR at 5 years. Therefore, it is possible that the results we are seeing in our study may be due to patient non-compliance in the radiation omission cohort. For instance, fewer than half of patients with hormone-sensitive disease in the radiation omission cohort took adjuvant endocrine therapy. However, even in the specific subgroup of patients with hormone-sensitive disease that received endocrine therapy, radiation omission remained associated with worse OS. While all patients were classified as having received adjuvant chemotherapy and anti-HER2 therapy, the NCDB does not capture the number of cycles delivered—therefore, it is possible that the patients in the radiation omission group did not receive the full prescribed course of adjuvant systemic therapy. Certainly, suboptimal delivery of systemic therapy and radiation omission could explain the difference in OS between the two cohorts.

However, since there are currently no reported data regarding radiation omission in stage I, HER2+ BC after lumpectomy and given the high risk of LRR in HER2+ BC in the absence of anti-HER2 based systemic therapy, we must consider alternative hypotheses for the observed differences in OS in this study. One potential explanation for the negative effect of radiation omission on OS is that a significant interaction exists between anti-HER2 therapy and radiation. For instance, Kyndi et al.^35^ demonstrated that ER−/HER2+ patients with node-positive BC had high risks of LRR when postmastectomy radiation (PMRT) was given without trastuzamab. When trastuzumab and PMRT were given together, Tseng et al.^36^ found that PMRT was independently associated with an 88% reduction in LRR suggesting a synergistic effect. Finally, in patients with stage I–III HER2+ BC treated with mastectomy and adjuvant trastuzumab ± PMRT, Lanning et al. reported a trend toward higher risk of LRR without PMRT^37^. These studies suggest that caution must be taken into account when considering radiation omission in patients with HER2+ BC due to the potential synergistic effect of anti-HER2 therapy and radiation.

Another important consideration is to more closely examine the HER2+ populations that were less likely to receive adjuvant radiation. These included older patients with advanced age (≥70 years old) and comorbidities as well as socioeconomic factors such as Hispanic ethnicity and income. While there is precedent for radiation omission in elderly patients with T1N0 ER+/HER2− BC, we do not yet have data that this is a safe approach in elderly women with HER2+ disease. Radiation omission in HER2+ BC could translate into higher risk of developing distant metastases and death from BC given that the recurrence risk peaks early in the disease course, particularly for ER-/PR-/HER2+ disease, and then plateaus^38^. This is unlike the case for ER+/HER2− BC where the risk of recurrence is low and constant over time^38^. Therefore, until there are mature data demonstrating the safety of radiation omission in T1N0 HER2+ BC, strong consideration should be given for post-lumpectomy irradiation and utilization of hypofractionated delivery methods to minimize burden of care.

We found that Hispanic women were less likely to receive radiation relative to non-Hispanic white women. In a recent analysis of women with BC of all subtypes diagnosed from 2010 to 2011, Hispanic women with stage I or II ER+/HER2+ disease were 40% more likely to receive guideline discordant care compared to white women^39^. Our study is consistent in that we found Hispanic women were 50% more likely to not receive radiation compared to white women. Other studies have demonstrated that Black women are less likely to receive trastuzamab and less likely to complete a year-long course of trastuzumab relative to white women^40,41^. While we did not detect a similar pattern for radiation omission in Black women, the number of non-white patients was small, particularly in the radiation omission cohort.

In addition, we closely examined survival outcomes in the patients that did not receive radiation therapy in order to identify factors that might be associated with improved OS outcomes. The only factor associated with improved OS in this patient cohort was treatment group based on hormone-receptor status and receipt of endocrine therapy. Relative to patients with hormone-sensitive disease that did not receive endocrine therapy, patients with hormone-sensitive disease that did receive endocrine therapy and patients with ER-/PR- disease were associated with better survival outcomes. While de-escalation of radiation therapy would not be considered in T1N0 ER−/PR−/HER2+ disease, the more biologically favorable group of patients with T1N0 hormone-sensitive HER2+ disease that are committed to taking endocrine therapy represents a population in which radiation de-escalation may be considered. However, in our propensity score matched analysis in this subgroup of patients, we found that radiation omission remained associated with worse OS.

An alternative to radiation omission may be APBI, which delivers treatment to the tumor bed region alone in 5–10 days. The proportion of patients with HER2+ disease treated with APBI was low (~5–6%) or unknown in the two largest APBI trials^11,12^. On the APT trial, only 4% of patients treated with lumpectomy received APBI. The potential advantages of APBI include reducing the volume of breast tissue exposed to radiation and shortening the treatment course to 5 days. APBI delivered with anti-HER2 therapy in the adjuvant setting could serve as a first step in de-escalation of radiation while additional evidence evaluation radiation omission in early stage, HER2+ BC are collected.

Our study has several limitations. The NCDB does not capture data on LRR and/or cancer-specific survival. Therefore, it is unclear if the higher mortality seen in the radiation omission group is due to recurrent disease vs. patient non-compliance with recommend treatments vs. treatment-related toxicity vs. other significant comorbidities particularly with a <30 month median follow-up. While we were able to control and match for known covariates included in the NCDB, other selection biases that influence radiation omission likely persist and could exaggerate the difference in OS seen between the groups. As previously mentioned, the radiation omission group may mask a non-compliant patient cohort that also did not receive adequate systemic therapy, which would place these patients at higher risk of recurrence and death from BC. The NCDB does not provide the end dates of systemic therapy or number of cycles delivered, so it is not possible to capture whether the patients in the radiation omission group received the intended course of systemic therapy. In addition, we are unable to further investigate the reasons why 234 patients in the radiation omission group were classified as not having radiation planned as part of their treatment course when there are no data available to suggest that it is safe to omit adjuvant radiation in HER2+ disease post-lumpectomy. However, we did confirm that there were no differences in the characteristics of patients in which radiation was not planned as part of their course compared to those that refused radiation therapy. There is an ongoing study of radiation omission in HER2+ patients achieving a pathologic complete response to neoadjuvant systemic therapy, but this would not be applicable to the patient population we studied^42^. In addition, the NCDB does not give details on the specific chemotherapy and anti-HER2 therapy agents used nor the duration of use. Nearly, two-thirds of patients were categorized as having received multi-agent chemotherapy, and it is unclear if this low-risk population was treated with multi-agent chemotherapy regimens (doxorubicin, cyclophosphamide, paclitaxel, and trastuzamab; docetaxel/paclitaxel/trastuzumab, etc.) or if these patients received paclitaxel/trastuzumab and were mistakenly classified as having received multi-agent chemotherapy. Since the APT trial was published in 2015, it is quite possible that multi-agent chemotherapy regimens were used during 2013–2015 which means that RT omission in patients that receive single-agent chemotherapy (paclitaxel/trastuzumab) could result in worse OS outcomes.

In conclusion, we found that radiation omission is independently associated with an increased risk of death in patients with T1N0 HER2+ BC treated with lumpectomy, adjuvant chemotherapy and anti-HER2 therapy. Patients that did not receive radiation tended to be older with more comorbidities, to be Hispanic, and to live in lower income areas. While other selection biases that influence radiation omission likely persist, including the possibility that patients in this cohort were not compliant with systemic therapy, these data should give caution to radiation omission in T1N0 HER2+ BC.

## Methods

### Patient selection

We retrospectively reviewed the NCDB, which is a combined effort of the Commission on Cancer (CoC) of the American College of Surgeons and the American Cancer Society. The NCDB is a nationwide hospital-based database that contains de-identified hospital registry data from more than 1500 accredited facilities and represents more than 70% of newly diagnosed cancer cases in the United States^43^. The NCDB collects data on patient demographics and comorbidities, tumor characteristics and staging details, primary therapies administered, and overall survival. The CoC’s NCDB and the hospitals participating in the CoC NCDB are the source of the de-identified data and have not verified and are not responsible for the statistical validity of the data analysis nor the conclusions presented in this study. Since all data are de-identified, this study was deemed exempt by the Ohio State University Medical Center’s Cancer Institutional Review Board.

Patients diagnosed with T1N0 HER2+ BC from 2013 to 2015 were collected from the NCDB participant user file (2013 was the first year in which anti-HER2 therapies were classified as “Immunotherapy” and not “Chemotherapy” in the NCDB). We included patients treated with lumpectomy, adjuvant chemotherapy and anti-HER2 therapy. Mastectomy patients and those undergoing preoperative systemic therapy were excluded. Additional inclusion/exclusion criteria are summarized in Fig. 1.

### Treatment definitions

We defined two cohorts of patients: radiation omission and radiation received. We collected details of radiation treatments in the patients that received radiation and reasons radiation was not delivered in the omission group.

### Study variables

Age was measured as a continuous and categorical variable (<70 years old vs. ≥70 years old). Categorical variables included: Charlson–Deyo Score, measure of comorbidity (0 vs. ≥1 comorbidities); tumor laterality (left vs. right); hormone-receptor status: ER−/PR− vs. ER+ and/or PR+ (hormone-sensitive); tumor size (≤1 cm vs. >1 cm); tumor grade (grade 3 vs. grade 1–2 vs. unknown); facility type (academic vs. other vs. unknown); race/ethnicity (non-Hispanic White vs. non-Hispanic Black vs. Hispanic vs. Other vs. Unknown); household income status based on zip code (<$46,000/yr vs. ≥$46,000/year vs. unknown), and education level of community based on zip code (≥20% adults without high school diploma vs. <20% vs. unknown). Chemotherapy receipt was categorized as single-agent, multi-agent, or unknown. In hormone-sensitive patients, receipt of endocrine therapy was also captured. Distance to treatment facility was dichotomized by the median value for the entire cohort (8.9 miles) into ≥8.9 miles vs. <8.9 miles.

### Statistical methods

First, we evaluated factors associated with radiation omission. Differences in baseline study variables between patients the groups were evaluated with the chi-square test (categorical variables) or *t* test (continuous variables). Multivariable logistic regression was used to identify independent factors associated with radiation omission (variables with *p* < 0.20 on univariate analysis were included in the multivariate logistic regression model).

Second, we compared OS between the radiation omission and radiation delivered treatment groups. We hypothesized that the OS between the two treatment groups would be statistically similar. We evaluated OS by the Kaplan–Meier method and by a multivariate Cox proportional hazards model (including all variables with *p* < 0.20 on univariate analysis). For the analysis of OS in the entire cohort, hormone-receptor status and receipt of endocrine therapy were combined to form three groups of patients: Hormone-sensitive that received endocrine therapy; Hormone-sensitive that did not receive endocrine therapy; and ER−/PR−. In an additional subgroup analysis, we evaluated Cox proportional hazards models specifically in the ER−/PR− patients and hormone-sensitive patients.

In order to further minimize the effect of potential confounders, we used a propensity-score matched analysis. All baseline covariates mentioned in the study variables section were included in the propensity score model. Patients in the radiation omission group were matched to those in the radiation delivered group using a 1:1 nearest available match without replacement^44^ using a caliper size calculated as 20% of the standard deviation of the propensity score^45^. Common support of the propensity score distributions was evaluated graphically and balance was evaluated by computing the standardized difference of the covariates across groups^46^. Following PSM, OS between treatment groups was estimated using the Kaplan–Meier method and the effect of radiation omission was evaluated with a Cox proportional hazards model with robust standard errors to account for clustering in matched pairs. All statistical analyses were performed using SAS, version 9.4 (SAS Institute Inc., Cary, NC). A two-sided *p* value of <0.05 was considered statistically significant.

### Reporting summary

Further information on research design is available in the Nature Research Reporting Summary linked to this article.

## Supplementary information

Supplementary Materials

Reporting Summary Checklist

## Data Availability

The data that support the findings of this study are available from the National Cancer Database, but restrictions apply to the availability of these data, which are not publicly available. The original data from the National Cancer Data Base, as well as the derived analysis dataset, will be made available to researchers at the Commission on Cancer (CoC) centres, who have completed an application form and a Data Usage Agreement. Please contact NCDB_PUF@facs.org for data access requests. The data generated and analyzed during this study are described in the following metadata record: 10.6084/m9.figshare.13636853^47^.

## Code availability

All statistical analysis were conducted using SAS v9.4. The SAS code used for this analysis is available upon request. Please contact the corresponding author.

## References

[1] Fisher B (2002). Twenty-year follow-up of a randomized trial comparing total mastectomy, lumpectomy, and lumpectomy plus irradiation for the treatment of invasive breast cancer. N. Engl. J. Med..

[2] Poggi MM (2003). Eighteen-year results in the treatment of early breast carcinoma with mastectomy versus breast conservation therapy: the National Cancer Institute Randomized Trial. Cancer.

[3] van Dongen JA (2000). Long-term results of a randomized trial comparing breast-conserving therapy with mastectomy: European Organization for Research and Treatment of Cancer 10801 trial. J. Natl. Cancer Inst..

[4] Veronesi U (2002). Twenty-year follow-up of a randomized study comparing breast-conserving surgery with radical mastectomy for early breast cancer. N. Engl. J. Med..

[5] Haviland JS (2013). The UK Standardisation of Breast Radiotherapy (START) trials of radiotherapy hypofractionation for treatment of early breast cancer: 10-year follow-up results of two randomised controlled trials. Lancet Oncol..

[6] Whelan TJ (2010). Long-term results of hypofractionated radiation therapy for breast cancer. N. Engl. J. Med..

[7] group FT (2011). First results of the randomised UK FAST Trial of radiotherapy hypofractionation for treatment of early breast cancer (CRUKE/04/015). Radiother. Oncol..

[8] Murray Brunt A (2020). Hypofractionated breast radiotherapy for 1 week versus 3 weeks (FAST-Forward): 5-year efficacy and late normal tissue effects results from a multicentre, non-inferiority, randomised, phase 3 trial. Lancet.

[9] Meattini I (2020). Abstract GS4-06: accelerated partial breast or whole breast irradiation after breast conservation surgery for patients with early breast cancer: 10-year follow up results of the APBI IMRT Florence randomized phase 3 trial. Cancer Res..

[10] Strnad V (2016). 5-year results of accelerated partial breast irradiation using sole interstitial multicatheter brachytherapy versus whole-breast irradiation with boost after breast-conserving surgery for low-risk invasive and in-situ carcinoma of the female breast: a randomised, phase 3, non-inferiority trial. Lancet.

[11] Vicini FA (2019). Long-term primary results of accelerated partial breast irradiation after breast-conserving surgery for early-stage breast cancer: a randomised, phase 3, equivalence trial. Lancet.

[12] Whelan TJ (2019). External beam accelerated partial breast irradiation versus whole breast irradiation after breast conserving surgery in women with ductal carcinoma in situ and node-negative breast cancer (RAPID): a randomised controlled trial. Lancet.

[13] Smith BD (2018). Radiation therapy for the whole breast: executive summary of an American Society for Radiation Oncology (ASTRO) evidence-based guideline. Pract. Radiat. Oncol..

[14] Hughes KS (2013). Lumpectomy plus tamoxifen with or without irradiation in women age 70 years or older with early breast cancer: long-term follow-up of CALGB 9343. J. Clin. Oncol..

[15] Kunkler IH (2015). Breast-conserving surgery with or without irradiation in women aged 65 years or older with early breast cancer (PRIME II): a randomised controlled trial. Lancet Oncol..

[16] ISRCTN41579286. PRIMETIME—Post-operative avoidance of radiotherapy in minimal risk women: patient selection using biomarkers. 10.1186/ISRCTN41579286 (2020).

[17] NCT01791829. A Prospective Cohort Study Evaluating Risk of Local Recurrence Following Breast Conserving Surgery and Endocrine Therapy in Low Risk Luminal A Breast Cancer (LUMINA). https://clinicaltrials.gov/ct2/show/NCT01791829 (2020).

[18] NCT02400190. The IDEA Study (Individualized Decisions for Endocrine Therapy Alone). https://clinicaltrials.gov/ct2/show/NCT02400190 (2020).

[19] NCT02653755. The PRECISION Trial (Profiling Early Breast Cancer for Radiotherapy Omission): A Phase II Study of Breast-Conserving Surgery Without Adjuvant Radiotherapy for Favorable-Risk Breast Cancer. https://www.clinicaltrials.gov/ct2/show/NCT02653755 (2020).

[20] NCT02889874. EXamining PErsonalised Radiation Therapy for Low-risk Early Breast Cancer (EXPERT). https://clinicaltrials.gov/ct2/show/NCT02889874 (2020).

[21] NCT03646955. Partial Breast Versus no Irradiation for Women With Early Breast Cancer. https://clinicaltrials.gov/ct2/show/NCT03646955 (2020).

[22] Yin W, Jiang Y, Shen Z, Shao Z, Lu J (2011). Trastuzumab in the adjuvant treatment of HER2-positive early breast cancer patients: a meta-analysis of published randomized controlled trials. PLoS ONE.

[23] Tolaney SM (2015). Adjuvant paclitaxel and trastuzumab for node-negative, HER2-positive breast cancer. N. Engl. J. Med..

[24] Tolaney SM (2019). Seven-year follow-up analysis of adjuvant paclitaxel and trastuzumab trial for node-negative, human epidermal growth factor receptor 2-positive breast cancer. J. Clin. Oncol..

[25] Bellon JR (2019). Local-regional recurrence in women with small node-negative, HER2-positive breast cancer: results from a prospective multi-institutional study (the APT trial). Breast Cancer Res. Treat..

[26] Tolaney, S. M. et al. TBCRC 033: A randomized phase II study of adjuvant trastuzumab emtansine (T-DM1) vs paclitaxel (T) in combination with trastuzumab (H) for stage I HER2-positive breast cancer (BC) (ATEMPT) [abstract]. In *Proceedings of the 2019 San Antonio Breast Cancer Symposium* Antonio, TX. Philadelphia (PA): AACR; *Cancer Res*. **80**, Abstract nr GS1-05, (2020).

[27] Bellon JR (2015). Personalized radiation oncology for breast cancer: the new frontier. J. Clin. Oncol..

[28] Fiorentino A (2015). Personalized-not omitted-radiation oncology for breast cancer. J. Clin. Oncol..

[29] Early Breast Cancer Trialists’ Collaborative, G. (2011). Effect of radiotherapy after breast-conserving surgery on 10-year recurrence and 15-year breast cancer death: meta-analysis of individual patient data for 10,801 women in 17 randomised trials. Lancet.

[30] Jayasekera J (2018). Effects of radiotherapy in early-stage, low-recurrence risk, hormone-sensitive breast cancer. J. Natl. Cancer Inst..

[31] Mamounas EP (2010). Association between the 21-gene recurrence score assay and risk of locoregional recurrence in node-negative, estrogen receptor-positive breast cancer: results from NSABP B-14 and NSABP B-20. J. Clin. Oncol..

[32] Potter R (2007). Lumpectomy plus tamoxifen or anastrozole with or without whole breast irradiation in women with favorable early breast cancer. Int J. Radiat. Oncol. Biol. Phys..

[33] Arvold ND (2011). Age, breast cancer subtype approximation, and local recurrence after breast-conserving therapy. J. Clin. Oncol..

[34] Jayasekera J (2018). Simulation modeling of cancer clinical trials: application to omitting radiotherapy in low-risk breast cancer. J. Natl. Cancer Inst..

[35] Kyndi M (2008). Estrogen receptor, progesterone receptor, HER-2, and response to postmastectomy radiotherapy in high-risk breast cancer: the Danish Breast Cancer Cooperative Group. J. Clin. Oncol..

[36] Tseng YD (2015). Biological subtype predicts risk of locoregional recurrence after mastectomy and impact of postmastectomy radiation in a large national database. Int J. Radiat. Oncol. Biol. Phys..

[37] Lanning RM (2015). The effect of adjuvant trastuzumab on locoregional recurrence of human epidermal growth factor receptor 2-positive breast cancer treated with mastectomy. Ann. Surg. Oncol..

[38] Cossetti RJ, Tyldesley SK, Speers CH, Zheng Y, Gelmon KA (2015). Comparison of breast cancer recurrence and outcome patterns between patients treated from 1986 to 1992 and from 2004 to 2008. J. Clin. Oncol..

[39] Chen L, Li CI (2015). Racial disparities in breast cancer diagnosis and treatment by hormone receptor and HER2 status. Cancer Epidemiol. Biomark. Prev..

[40] Freedman RA (2013). Use of adjuvant trastuzumab in women with human epidermal growth factor receptor 2 (HER2)-positive breast cancer by race/ethnicity and education within the National Comprehensive. Cancer Netw. Cancer.

[41] Reeder-Hayes K, Peacock Hinton S, Meng K, Carey LA, Dusetzina SB (2016). Disparities in use of human epidermal growth hormone receptor 2-targeted therapy for early-stage breast cancer. J. Clin. Oncol..

[42] NCT03460067. Omission of Radiation in Patients With Her-2 Positive Breast Cancer. https://clinicaltrials.gov/ct2/show/NCT03460067 (2020).

[43] Bilimoria KY, Stewart AK, Winchester DP, Ko CY (2008). The National Cancer Data Base: a powerful initiative to improve cancer care in the United States. Ann. Surg. Oncol..

[44] Coca-Perraillon, M. Local and global optimal propensity score matching. in *SAS Global Forum 2007 Conference* (Orlando, FL, 2007).

[45] Rosenbaum PR, Rubin DB (1985). Constructing a control-group using multivariate matched sampling methods that incorporate the propensity score. Am. Stat..

[46] Lanehart, R. E., et al. Propensity score analysis and assessment of propensity score approaches using SAS procedures. in *SAS Global Forum 2012 Conference* (Orlando, FL, 2012).

[47] Bazan, J. G. et al. Metadata supporting the article: de-escalation of radiation therapy in patients with stage I, node-negative, HER2-positive breast cancer. *figshare*10.6084/m9.figshare.13636853 (2021). 10.1038/s41523-021-00242-8 PMC7994398 33767168

