# Supplementary material for: De-escalation of radiation therapy in patients with stage I, node-negative, HER2-positive breast cancer
Source: NPJ Breast Cancer. 2021 Mar 25;7:33. doi: 10.1038/s41523-021-00242-8 (PMC7994398; doi:10.1038/s41523-021-00242-8)
Supplement: Supplementary file 1 — Supplementary Materials [file 41523_2021_242_MOESM1_ESM.pdf]

SUPPLEMENTARY TABLES

Supplementary Table 1: Patient and tumor characteristics in patients that refused radiation therapy compared to patients in which radiation was not planned as their initial treatment course.

	Refused RT (N=254)	RT Not Planned (N=234)	p-value
**Age**			
Mean (standard deviation)	64.3 (11.2)	63.8 (12.1)	0.6496
≥70 yrs, N (%)	105 (41.3%)	98 (41.95)	0.9035
**Comorbidities**			0.1008
0	193 (76.0%)	192 (82.0%)	
≥1	61 (24.0%)	42 (18.0%)	
**Laterality**			0.7004
Left	126 (49.6%)	112 (47.9%)	
Right	128 (50.4%)	122 (52.1%)	
**Hormone Status**			0.6874
ER+ or PR+	187 (73.6%)	176 (75.2%)	
ER-/PR-	67 (26.4%)	58 (24.8%)	
**Tumor Size**			0.3999
≤1 cm	82 (32.3%)	84 (35.9%)	
>1-2 cm	172 (67.7%)	150 (64.1%)	
**Tumor Grade**			0.7701
Grade 3	132 (52.0%)	115 (49.2%)	
Grade 1-2	109 (42.9%)	108 (46.1%)	
Unknown	13 (5.1%)	11 (4.7%)	
**Facility Type**			0.5206
Academic	80 (31.5%)	64 (27.4%)	
Other	167 (65.8%)	165 (70.5%)	
Unknown	7 (2.7%)	5 (2.1%)	
**Race/Ethnicity**			0.2509
Non-Hispanic White	189 (74.4%)	189 (80.8%)	
Non-Hispanic Black	29 (11.4%)	19 (8.1%)	
Hispanic	21 (8.3%)	10 (4.3%)	
Non-Hispanic Other	13 (5.1%)	13 (5.6%)	
Unknown	2 (0.8%)	3 (1.2%)	
**Income Status**			0.8734
<$46,000/yr	143 (56.3%)	133 (56.8%)	
≥$46,000/yr	100 (39.4%)	93 (39.7%)	
Unknown	11 (4.3%)	8 (3.4%)	
**No HSD**			0.1245
≥20%	101 (39.8%)	74 (31.6%)	
<20%	142 (55.9%)	152 (65.0%)	
Unknown	11 (4.3%)	8 (3.4%)	
**Distance to Facility**			0.4594
≥8.9 miles	125 (49.2%)	123 (52.6%)	
<8.9 miles	129 (50.8%)	111 (47.4%)	
**Chemotherapy**			0.3646
Single-Agent	99 (39.0%)	95 (40.6%)	
Multi-Agent	151 (59.5%)	131 (56.0%)	

Not Specified	4 (1.5%)	8 (3.4%)	
**Receipt of ET**[1]			0.1772
Yes	81 (31.9%)	93 (39.7%)	
No	106 (41.7%)	83 (35.5%)	

Abbreviations: RT=radiation therapy; SD=standardized difference; NHW=non-Hispanic white; NHB=non-Hispanic Black; HSD=High-school diploma; [1]Only in patients with ER+ and/or PR+ disease

Supplementary Table 2: Characteristics of the propensity-matched cohort in patients with hormone-sensitive breast cancer that received endocrine therapy.

	Received RT (N=182)	RT omission (N=182)	Standardized Difference
**Age**			
Mean (SD)	64.4 (11.1)	64.8 (11.7)	0.0387
≥70 yrs, N (%)	81 (44.5%)	82 (45.1%)	0.0110
**Comorbidities**			0.0412
0	147 (80.8%)	144 (79.1%)	
≥1	35 (19.2%)	38 (20.9%)	
**Laterality**			0.1219
Left	107 (58.8%)	96 (52.8%)	
Right	75 (41.2%)	86 (47.3%)	
**Tumor Size**			0.0594
≤1 cm	48 (26.4%)	47 (25.8%)	
>1-2 cm	134 (73.6%)	135 (74.2%)	
**Tumor Grade**			0.0583
Grade 3	73 (40.1%)	77 (42.3%)	
Grade 1-2	97 (53.3%)	95 (52.2%)	
Unknown	12 (6.6%)	10 (5.5%)	
**Facility Type**			0.0628
Academic	54 (29.7%)	55 (30.2%)	
Other	127(69.8%)	125 (68.7%)	
Unknown	1 (0.5%)	2 (1.1%)	
**Race/Ethnicity**			0.0158
NH-White	154 (84.6%)	149 (81.9%)	
NH-Black	15 (8.2%)	14 (7.7%)	
Hispanic	4 (2.2%)	8 (4.4%)	
NH-Other	9 (5.0%)	10 (5.5%)	
Unknown	0 (0%)	1 (0.5%)	
**Income Status**			0.0341
<$46,000/yr	116 (63.7%)	113 (62.1%)	
≥$46,000/yr	66 (36.3%)	69 (37.9%)	
Unknown	0 (0%)	0 (0%)	
**No HSD**			0.0581
≥20%	66 (36.3%)	61 (33.5%)	
<20%	111 (61.0%)	116 (63.8%)	
Unknown	5 (2.7%)	5 (2.7%)	
**Distance to Facility**			
≥8.9 miles	90 (49.5%)	89 (48.9%)	0.0110
<8.9 miles	92 (50.5%)	93 (51.1%)	
**Chemotherapy**			0.0110
Single-agent	80 (44.0%)	79 (43.4%)	
Multi-agent	101 (55.5%)	100 (55.0%)	
Not specified	1 (0.5%)	3 (1.6%)	

Abbreviations: RT=radiation therapy; SD=standard deviation; NHW=non-Hispanic white; NHB=non-Hispanic Black; HSD=High-school diploma.

Supplementary Figure 1: Overall survival of patients based on hormone-receptor status and receipt of endocrine therapy.

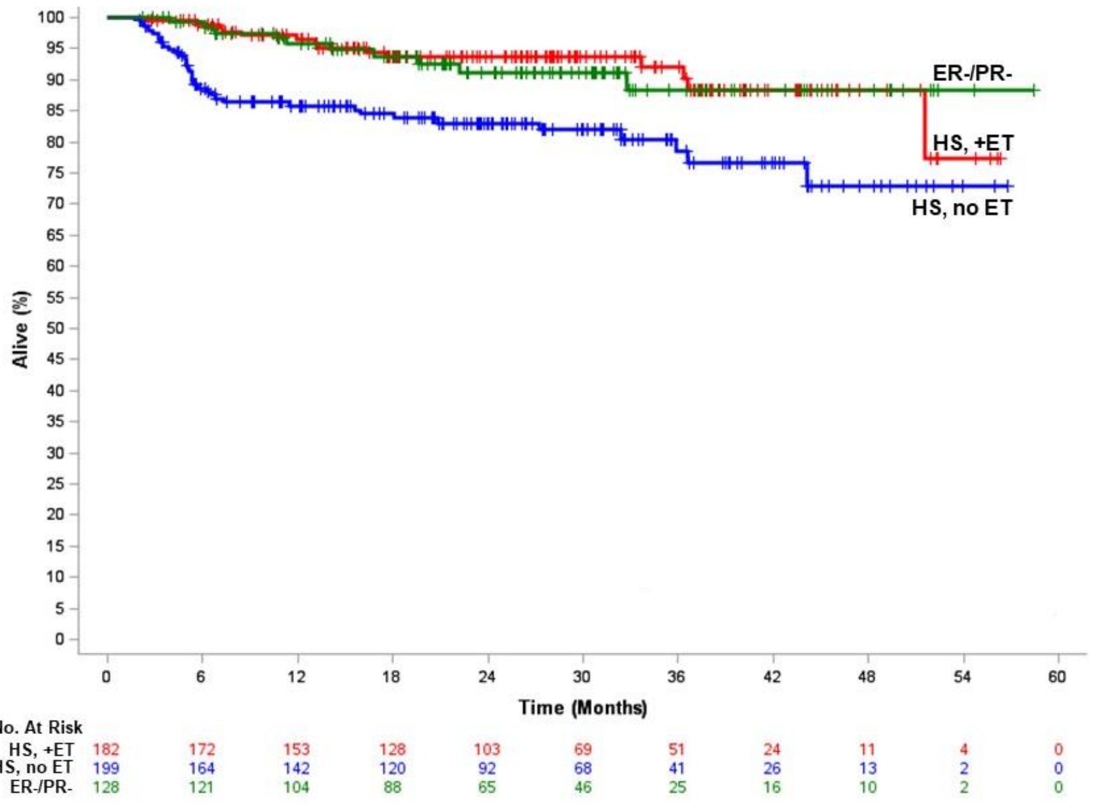

Abbreviations: HS=hormone-sensitive; ET=endocrine therapy.
