# Supplementary material for: De-escalation of radiation therapy in patients with stage I, node-negative, HER2-positive breast cancer
Source: NPJ Breast Cancer. 2021 Mar 25;7:33. doi: 10.1038/s41523-021-00242-8 (PMC7994398; doi:10.1038/s41523-021-00242-8)
Supplement: Supplementary file 2 — Reporting Summary Checklist [file 41523_2021_242_MOESM2_ESM.pdf]

nature research

Corresponding author(s): Jose G. Bazan

Last updated by author(s): 2021-28-02

Reporting Summary

Nature Research wishes to improve the reproducibility of the work that we publish. This form provides structure for consistency and transparency in reporting. For further information on Nature Research policies, see our Editorial Policies and the Editorial Policy Checklist.

Statistics

For all statistical analyses, confirm that the following items are present in the figure legend, table legend, main text, or Methods section.

n/a	Confirmed	
☐	☒	The exact sample size (n) for each experimental group/condition, given as a discrete number and unit of measurement
☒	☐	A statement on whether measurements were taken from distinct samples or whether the same sample was measured repeatedly
☐	☒	The statistical test(s) used AND whether they are one- or two-sided<br>*Only common tests should be described solely by name; describe more complex techniques in the Methods section.*
☐	☒	A description of all covariates tested
☐	☒	A description of any assumptions or corrections, such as tests of normality and adjustment for multiple comparisons
☐	☒	A full description of the statistical parameters including central tendency (e.g. means) or other basic estimates (e.g. regression coefficient) AND variation (e.g. standard deviation) or associated estimates of uncertainty (e.g. confidence intervals)
☐	☒	For null hypothesis testing, the test statistic (e.g. F, t, r) with confidence intervals, effect sizes, degrees of freedom and P value noted<br>*Give P values as exact values whenever suitable.*
☒	☐	For Bayesian analysis, information on the choice of priors and Markov chain Monte Carlo settings
☒	☐	For hierarchical and complex designs, identification of the appropriate level for tests and full reporting of outcomes
☐	☒	Estimates of effect sizes (e.g. Cohen's d, Pearson's r), indicating how they were calculated

Our web collection on statistics for biologists contains articles on many of the points above.

Software and code

Policy information about availability of computer code

Data collection	SAS version 9.4 was used to open the data set file provided by the National Cancer Database.
Data analysis	All statistical analyses were performed using SAS version 9.4.

For manuscripts utilizing custom algorithms or software that are central to the research but not yet described in published literature, software must be made available to editors and reviewers. We strongly encourage code deposition in a community repository (e.g. GitHub). See the Nature Research guidelines for submitting code & software for further information.

Data

Policy information about availability of data

All manuscripts must include a data availability statement. This statement should provide the following information, where applicable:

- Accession codes, unique identifiers, or web links for publicly available datasets
- A list of figures that have associated raw data
- A description of any restrictions on data availability

The dataset from the National Cancer Database is publicly available with permission.

Field-specific reporting

Please select the one below that is the best fit for your research. If you are not sure, read the appropriate sections before making your selection.

☐ Life sciences ☒ Behavioural & social sciences ☐ Ecological, evolutionary & environmental sciences

For a reference copy of the document with all sections, see nature.com/documents/nr-reporting-summary-flat.pdf

Behavioural & social sciences study design

All studies must disclose on these points even when the disclosure is negative.

Study description	Data are quantitative. This is a retrospective cohort study.
Research sample	Breast cancer patients from the National Cancer Database. The NCDB encompasses approximately 70% of all newly diagnosed cancer cases in the United States.
Sampling strategy	We used specific criteria to identify patients for this retrospective cohort study. Patients had to have stage I (T1N0), HER2+ breast cancer that was treated with lumpectomy and both adjuvant chemotherapy AND anti-HER2 therapy.
Data collection	The dataset was provided by the National Cancer Database.
Timing	The collection dates used for this study were from 1/1/2013-12/31/2015.
Data exclusions	Patients in the NCDB that were treated prior to 2013 were excluded from this analysis. 2013 was the first year in which anti-HER2 therapy was classified as "Immunotherapy" in the NCDB and not "Chemotherapy." Therefore, patients that were treated prior to 2013 were not eligible for this study since we could not ascertain whether or not they received anti-HER2 based therapy. Additionally, patients that did not have T1N0, HER2+ disease were excluded from this analysis. Patients that were treated with mastectomy were excluded. Patients that received chemotherapy prior to surgery were excluded. Patients that did not receive adjuvant chemotherapy were excluded. Patients that did not receive adjuvant anti-HER2 therapy were excluded. All inclusion/exclusion criteria with the numbers excluded are provided in Figure 1 of the manuscript.
Non-participation	N/A - retrospective study
Randomization	N/A - retrospective study

Reporting for specific materials, systems and methods

We require information from authors about some types of materials, experimental systems and methods used in many studies. Here, indicate whether each material, system or method listed is relevant to your study. If you are not sure if a list item applies to your research, read the appropriate section before selecting a response.

Materials & experimental systems

n/a	Involved in the study	
☒	☐	Antibodies
☒	☐	Eukaryotic cell lines
☒	☐	Palaeontology and archaeology
☒	☐	Animals and other organisms
☒	☐	Human research participants
☐	☒	Clinical data
☒	☐	Dual use research of concern

Methods

n/a	Involved in the study	
☒	☐	ChIP-seq
☒	☐	Flow cytometry
☒	☐	MRI-based neuroimaging

Clinical data

Policy information about clinical studies

All manuscripts should comply with the ICMJE guidelines for publication of clinical research and a completed CONSORT checklist must be included with all submissions.

Clinical trial registration	Not applicable
Study protocol	This is a retrospective study. All details are provided in the Methods section.
Data collection	Institutions that participate in the NCDB collect data locally and then report the data to the NCDB.

Outcomes

The primary outcome was overall survival. This was defined as the time from the date of diagnosis to the date of death or the date of last follow-up.
